# Results of the NIST National Ball Plate Round Robin

**DOI:** 10.6028/jres.102.008

**Published:** 1997

**Authors:** G. W. Caskey, S. D. Phillips, B. R. Borchardt

**Affiliations:** National Institute of Standards and Technology, Gaithersburg, MD 20899-0001

**Keywords:** ball plate, CMM, coordinate measuring machine, coordinate metrology, round robin

## Abstract

This report examines the results of the ball plate round robin administered by NIST. The round robin was part of an effort to assess the current state of industry practices for measurements made using coordinate measuring machines. Measurements of a two-dimensional ball plate (240 mm by 240 mm) on 41 coordinate measuring machines were collected and analyzed. Typically, the deviations of the reported *X* and *Y* coordinates from the calibrated values were within ± 5 μm, with some coordinate deviations exceeding 20.0 μm. One of the most significant observations from these data was that over 75 % of the participants failed to correctly estimate their measurement error on one or more of the ball plate spheres.

## 1. Introduction

This paper presents the final results, along with some analysis, of the ball plate round robin organized by the National Institute of Standards and Technology in cooperation with the National Conference of Standards Laboratories (NCSL) and the University of North Carolina at Charlotte (UNCC). The impetus behind this ball plate round robin was to provide a simple method for the assessment of the current state of industrial measurement capability using coordinate measuring machines (CMMs). Historically, round robins (where a single artifact is circulated among the participants for measurement) have been well suited for such a purpose. In particular, this round robin was modeled after the CIRP (International Institution for Production Engineering Research) international round robin for which a similar ball plate was sent to various national laboratories for measurement.

## 2. Round Robin Demographics

The round robin participants included various U.S. public and private manufacturing organizations that are engaged in coordinate metrology using CMMs. To provide a fair comparison, only computer controlled coordinate measuring machines were included in the study. Additionally, since the goal of this round robin was to assess industrial measurement practices, manufacturers of CMMs were excluded as participants because they have the advantage of routinely performing extensive characterizations of their machines.

There were a total of 16 organizations that volunteered to participate in this round robin, representing a substantial portion of the manufacturing spectrum. Most participants are leaders in their fields, which include aerospace, heavy equipment, petroleum equipment and defense facilities. From the 16 participants, ball plate measurement data on a total of 41 CMMs was collected and analyzed. These machines included models from many U.S. and foreign CMM manufacturers, and ranged from ultra precision to shop floor accuracy machines with axis lengths up to 2.5 m.

## 3. Ball Plate Description

**Fig. 1:**
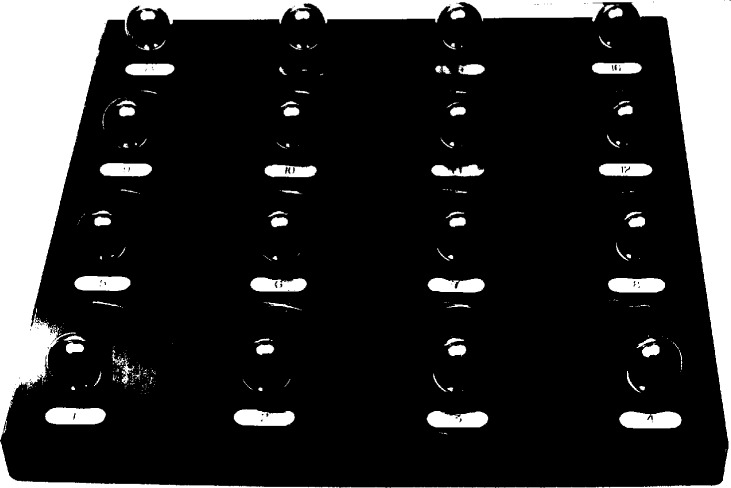
The ball plate used in the round robin. The spheres are numbered sequentially from left to right, by rows, beginning with number 1 in the lower left corner.

The ball plate used for this round robin, shown in Fig. 1, is a two-dimensional array of tool steel balls mounted on a base plate. Attached to the ball plate are 16 spheres equally spaced on an 80 mm by 80 mm grid. Nominal sphere center coordinates and their distance from sphere number 1 (the defined plate coordinate system origin) are given in Table 1. Each sphere is 25.4 mm (1 in) in diameter and is round to better than 0.15 μm. The base of the ball plate is mild carbon steel with three semikinematic pads ground on the bottom for mounting. One of the primary concerns was that the ball plate be constructed of a manageable size and weight to facilitate handling. At the same time, it was equally important that the plate be sufficiently robust to prevent permanent deformation due to normal handling. Therefore, the ball plate was limited to 240 mm by 240 mm, along its length and width, but was made relatively massive in thickness (25 mm) to accommodate these competing requirements.

The ball plate was chosen as the measurement artifact for this round robin due to its simple design and ease of measurement. Most coordinate measuring machine users are familiar with the measurement of spheres due to their widespread use as probe calibration artifacts. Although a more complicated artifact could have been chosen to better represent manufactured parts, it was felt that this type of artifact was a suitable choice for the broad spectrum of round robin participants. We point out that ball plate measurements provide only limited information about the CMM. In particular, since this plate was designed to be measured in the plane of the CMM table no information is determined regarding the vertical axis of the CMM, e.g., *Z* axis squareness or *Z* axis roll etc. Furthermore, since only the center-to-center distances between the spheres are reported many effects such as probe lobing or repeatability problems are averaged out in the sphere fitting process. Finally, the center-to-center distances are insensitive to errors in the effective size of the stylus, which would appear in actual feature size measurements such as the length of a gage block or the diameter of a sphere. Consequently, the ball plate results should be viewed as representing only a portion of the potential errors which could occur in an actual part measurement.

## 4. Measurement Procedures

The participants were asked to measure the plate and report the *X* and *Y* ball center coordinates along with the estimated errors in these coordinates. They were given the option of measuring the plate on multiple machines (if applicable) as long as each measurement was independently reported. The only instructions concerning the measurement process pertained to the reporting of the data and stated that the center of ball number 1 be defined as the origin of the plate coordinate system, and that the center of ball number 4 be defined to lie on the plate *X* axis. Additionally, it was requested that the ball center coordinates be reported referenced to the standard temperature of 20 °C [the nominal coefficient of thermal expansion for this plate was specified as 11.6 μm/(m? °C]). However, correcting for nominal differential thermal expansion was left to the discretion of the individual participant since this correction is part of the overall measurement process under assessment. There were no additional instructions on how the plate was to be measured—simply that the plate be treated as a high accuracy part. The measurement plan, such as the number of points per sphere, number of times each sphere was to be measured, the location and orientation of the plate measurement(s) within the machine measurement volume, probing parameters (probe approach rate, probe approach distance), etc., were also left to the discretion of the individual participant for the previously stated reason.

## 5. Results and Data Analysis

### 5.1 Ball Plate Calibration Values

The coordinate deviations reported in this paper are with respect to the calibrated values defined as the mean of the measurements made by NIST and UNCC on their respective coordinate measuring machines. The worst case difference from the mean of these baseline measurements was 0.5 μm with typical agreement better than 0.25 μm. Measurements were made on two different coordinate measuring machines using redundant measurement techniques [1,2], which effectively eliminate most of the residual CMM errors that would affect the measurement results for this type of artifact. Upon completion of the round robin, the ball plate was remeasured at NIST to assess the dimensional stability of the plate. This was necessary due to the rather extended period of time required to conduct the round robin (approximately 24 months from Spring 1992 to Spring 1994).

**Fig. 2:**
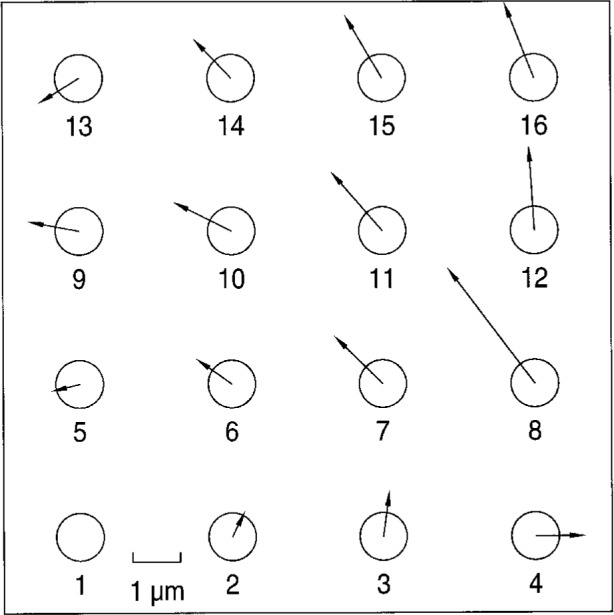
Graphical representation of shift in ball plate sphere centers. The maximum change in center coordinates, in excess of 3 µm, occurred on ball number 8.

During the remeasurement of the plate by NIST at the end of the round robin, it was discovered that there was an apparent systematic shift in sphere center. The magnitude and direction of the change in these coordinates, shown graphically in Fig. 2, is consistent with a distortion occurring predominately on the right side of the plate. The average magnitude of the ball center coordinate change in this area was approximately 2 μm, with a maximum change of more than 3 μm occurring at ball number 8.

In order to verify that there was a change in some of the sphere center coordinates and to narrow down the time at which this change occurred, the ball plate was sent to a participant who previously measured the plate around the midpoint of the round robin. This participant remeasured the ball on the same high accuracy CMM, but this time using the same redundant measurement techniques as NIST. The results from this remeasurement agreed well with the final set of NIST sphere center coordinates (while differing significantly from the participant’s previously submitted round robin measurement results). This confirmed that the ball plate had indeed changed sometime after the midpoint of the round robin.

By reanalyzing the participants’ data using both sets of NIST sphere center coordinates, it was determined that the plate change was a sudden rather than a gradual event (indicating probable damage to the plate through mishandling). Additionally, a more accurate determination of the time frame within which the change occurred was made. Subsequently, the data for those participants who measured the plate after this time were compared against the final set of NIST measurement coordinates, while the earlier participants’ data were compared with the calibrated values established from the mean of the NIST and UNCC measurement data.

### 5.2 Round Robin Results

**Fig. 3:**
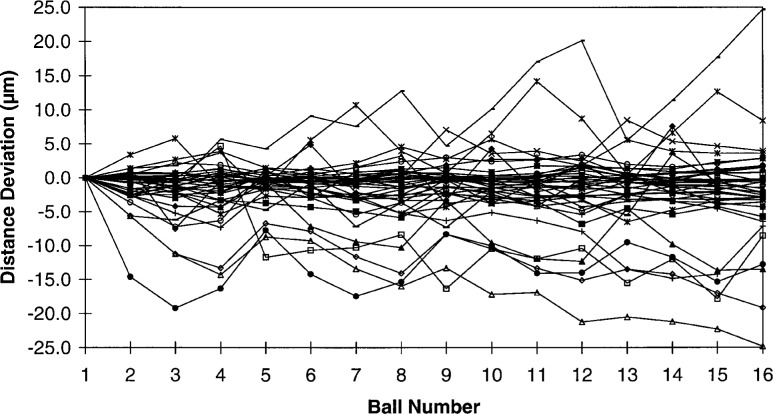
The deviations in the sphere distances, between ball number 1 and the remaining 15 balls, with respect to the NIST calibrated values.

The results of the ball plate round robin are shown in Figs. 3 through 7. Figure 3 shows the deviations in distance from ball number 1 (the ball plate measurement system origin) for all of the participants. The authors realize that choosing to analyze only the set of distances from ball number 1 can have the effect of biasing the individual participants’ deviation data since the measurement of ball number 1 is itself not without error. However, the prescribed procedure for establishing a common ball plate coordinate system was very much analogous to establishing a part coordinate system and/or part datums which are integral to the discrete part measurement process with a CMM. Therefore, analyzing the data in this way was more closely aligned with the objectives of the round robin.

**Fig. 4:**
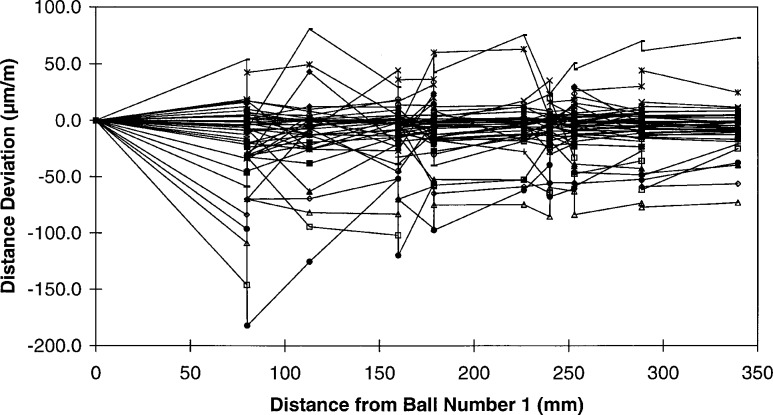
The distance deviations from Fig. 1 expressed as a fraction of the distance from ball number 1.

A similar plot, Fig. 4, shows these same distance deviations expressed as a fraction of each sphere’s distance from ball number 1. Analyzing the data in this manner provides an indication of how well the individual machines could measure two-dimensional features of various lengths. As evident from the plot, the majority of the machines are within ± 50 μm/m of feature length. Although those data are correct as presented, they are somewhat misleading over short distances where the non-repeatability of the CMM tends to be the predominate error source. In this regime the distance deviations expressed as a fraction of the feature length can be inflated since the non-repeatability effects remain constant even as the measurement length becomes small.

### 5.3 Participants’ Measurement Error Estimation

**Fig. 5:**
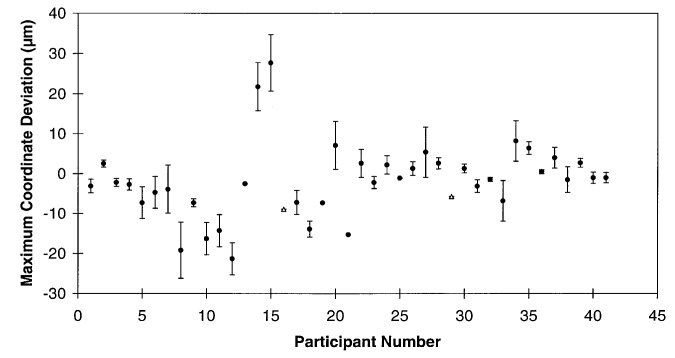
Maximum coordinate deviations (from the calibrated values) along with participants measurement error estimates. The deviations are represented by the solid circles and the error estimates by the error bars. Several participants did not provide an error estimate and their deviations are denoted by the triangles.

Data reporting for this round robin, by the participants, was supposed to include an estimate of the “measurement error” which was defined as 3 standard deviations of the observed measurements arithmetically added to any estimated systematic errors.^1^ Providing an estimate of their measurement error proved to be difficult for some of the participants, with responses varying from the estimates as requested to no estimates at all. Figure 5 shows the measurement error estimates (±) plotted around the maximum sphere center coordinate deviation reported by each participant. These deviations were chosen without regard to ball number or coordinate (*X* or *Y*).

It can be seen from this plot that the maximum deviations for a majority of the participants are within ± 10 μm. However, as was previously discussed there are additional error sources not revealed by the ball plate measurement results which can further degrade actual CMM performance. Therefore, it is somewhat disturbing that over 75 % of the participants failed to correctly estimate their measurement error on at least one of the ball plate spheres. (Figure 5 does not necessarily represent the worst case error estimation since the maximum coordinate deviation and the worst case error estimates need not occur on the same ball.) This further supports the call for more realistic CMM uncertainty estimation technique development by the CMM community as a whole.

### 5.4 Machine Error Examples

Ball plates have historically been used for the periodic performance evaluation and/or calibration of coordinate measuring machines [2,5]. The amount of information about the CMM that may be obtained from measurements of this type of artifact depends on the number and location of ball plate measurements, the suitability of the plate for mounting in the horizontal and vertical planes, and whether the ball plate is calibrated.

**Fig. 6:**
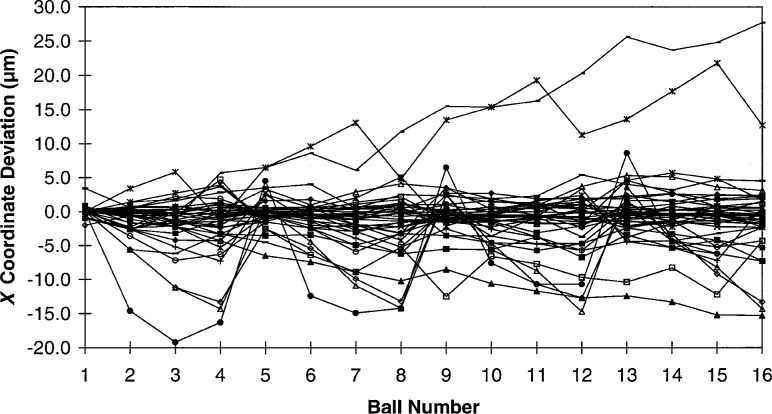
*X* coordinate deviations, from the calibrated values, for all round robin participants.

**Fig. 7:**
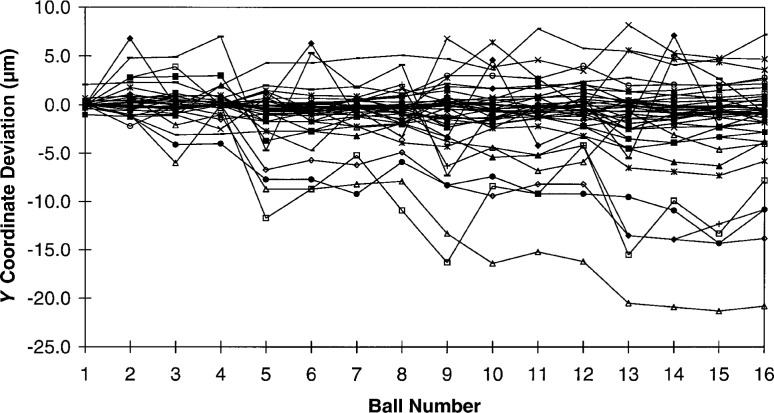
*Y* coordinate deviations, from the calibrated values, for all round robin participants.

**Fig. 8:**
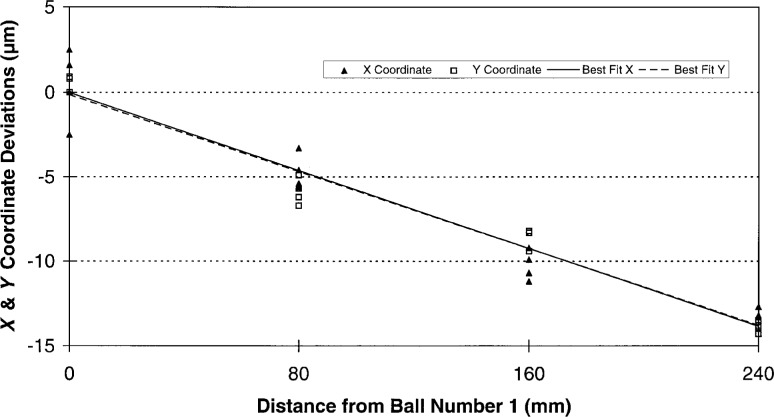
A thermal error indicated by one participant’s data, likely caused by improper thermal compensation of the CMM scales. The error here is approximately 54 µm/m indicating a temperature difference of 6 ° C to 7 ° C (∼11 ° F to 13 ° F).

Without imposing a rigorous measurement strategy on the participants, it would be difficult to deconvolve all of the individual errors for a series of independent ball plate measurements such as this round robin data. This is due to the highly interdependent nature of the these errors. However, we may examine the individual *X* and *Y* coordinate deviations looking for patterns in the data. As can be seen from Figs. 6 and 7, much of this data display a systematic structure, and in some cases, it is possible to identify one or more of the dominant errors. For instance, by comparing the *X* and *Y* coordinate deviations for a single set of measurements, it is possible to identify relative scale errors and/or thermal errors. If the deviations of the individual *X* and *Y* ball coordinates are approximately equal, as in Fig. 8, then a thermal error is suspect. This type of error could result from a failure to properly correct for ball plate expansion, CMM scale expansion, or both, when the measurements are made at a temperature other than the standard reference temperature (20 °C). The error shown in Fig. 8 is most likely the result of correcting the measurement data for ball plate thermal expansion while failing to correct for the CMM scale thermal expansion. This would account for the negative trend in the data, amounting to an error of approximately 54 μm/m indicating CMM scale temperatures of 26 °C to 27 °C (79 °F to 81 °F)—which is representative of many manufacturing facility environments.

**Fig. 9:**
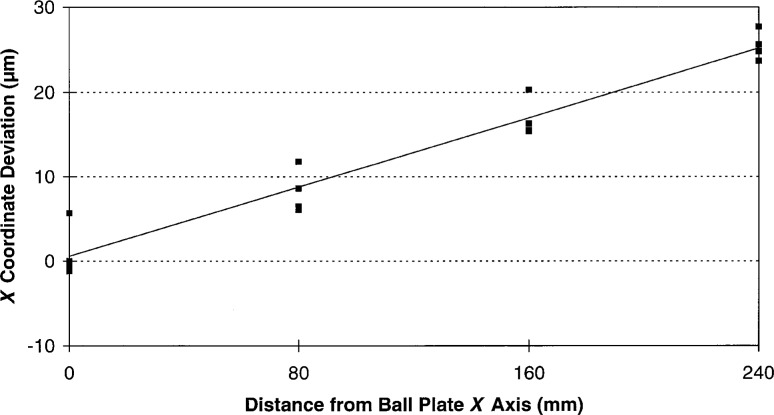
One participant’s data showing an out-of-squareness error. In this case the error is 100 μrad (approximately 20 arcseconds).

Another identifiable error is the out-of-squareness between the two measurement axes. Since the *X* axis of the ball plate was defined by the plate coordinate system, any squareness error would appear as an out-of-squareness of the *Y* axis with respect to the *X* axis. (The assignment of out-of-squareness to either of the axes is purely arbitrary as long as the magnitude, direction and effect on the measured coordinates is understood.) Therefore, by viewing the *X* deviation data in groups of nominally equal *Y* coordinates, any out-of-squareness between the *X* and *Y* axes will become apparent. Figure 9 shows evidence of a classical out-of-squareness error for one participant’s CMM. In this case the out-of-squareness is calculated as 100 microradians (approximately 20 arc seconds) of error.

Although we have presented the results from particular CMMs to illustrate these errors, similar geometry and/or thermal errors were evident in the data from many of the other CMMs in this round robin. The presence of such systematic errors indicates that more frequent testing of CMMs, i.e., statistical process control, may be needed. Comprehensive interim testing, a method of monitoring CMM performance in between regularly scheduled machine calibrations, is well suited to this task and is being widely adopted by both the national and internationalCMMstandard committees [6].

## 6. Summary

We have presented the final results of the NIST/NCSL/UNCC ball plate round robin. Ball plate measurement data on 41 different coordinate measuring machines from 16 different public and private industry participants were collected and analyzed. The data indicated that there was a shift in some of the sphere center coordinates, which occurred in the latter half of the round robin, apparently due to damage during handling or transit. This made it necessary to establish a second set of calibrated ball center coordinates against which the post damage participants’ data could be analyzed.

Comparing the participants’ data to the appropriate set of calibrated coordinates shows that a majority of the ball center deviation data was within ± 5 μm, with maximum deviations exceeding 20 μm. These deviations, expressed as a fraction of the measured feature length, were typically less than 25 μm/m. Simple analysis of the data revealed some of the possible sources of error that contributed to these deviations. Examples of temperature and squareness errors, taken from actual round robin data, were presented. An important part of this round robin exercise was the estimation, by the respective participants, of the measurement errors for their CMM. Of those participants that estimated the measurement errors, over 75 % exceeded this estimate on one or more of the spheres. This suggests that better methods for estimating measurement uncertainty (as detailed in the ISO *Guide to the Expression of Uncertainty in Measurement* [4]) combined with measurement process control, e.g., regular interim testing, are necessary to provide a higher level of confidence in measurements made using coordinate measuring machines.

## Figures and Tables

**Table 1:** Nominal sphere coordinates and distances from ball number 1

Ball number	*X* coordinate (mm)	*Y* coordinate (mm)	Distance from ball number 1 (mm)
1	0	0	0
2	80	0	80
3	160	0	160
4	240	0	240
5	0	80	80
6	80	80	113
7	160	80	178
8	240	80	252
9	0	160	160
10	80	160	178
11	160	160	226
12	240	160	288
13	0	240	240
14	80	240	252
15	160	240	288
16	240	240	339

## Footnotes

1 “Measurement error” as defined here is not consistent with current NIST policy [3,4] on the expression of measurement uncertainty. Under this policy, all quantifiable systematic errors would be corrected and the remaining uncertainties would then be estimated and combined into an expanded uncertainty with a coverage factor *k* = 2. However, this round robin was initiated prior to the adoption of this policy, so the interpretation of the results presented in this paper is consistent with the instructions provide to the participants.
